# Medium-throughput image-based phenotypic siRNA screen to unveil the molecular basis of B cell polarization

**DOI:** 10.1038/s41597-023-02301-0

**Published:** 2023-06-23

**Authors:** Dorian Obino, Mathieu Maurin, Florent Dingli, Damarys Loew, Aurianne Lescure, Emmanuel Terriac, Christel Goudot, Odile Malbec, Danielle Lankar, Maria-Isabel Yuseff, Ana-Maria Lennon-Duménil, Hélène D. Moreau

**Affiliations:** 1 Institut Curie, PSL Research University, Inserm U932, Immunity and Cancer, 75005 Paris, France; 2 Institut Curie, PSL Research University, CurieCoreTech Mass Spectrometry Proteomics, 75005 Paris, France; 3 Institut Curie, PSL Research University, Translational Research Department, BioPhenics Platform, PICT-IBISA, Paris, France; 4 Institut Curie, PSL Research University, CNRS UMR144 Paris, France; 5 Department of Cellular and Molecular Biology, Faculty of Sciences, Pontificia Universidad Católica de Chile, Santiago, Chile

**Keywords:** Apicobasal polarity, B cells, Antigen processing and presentation

## Abstract

Cell polarity is an essential and highly conserved process governing cell function. Cell polarization is generally triggered by an external signal that induces the relocation of the centrosome, thus defining the polarity axis of the cell. Here, we took advantage of B cells as a model to study cell polarity and perform a medium-throughput siRNA-based imaging screen to identify new molecular regulators of polarization. We first identified candidates based on a quantitative proteomic analysis of proteins differentially associated with the centrosome of resting non-polarized and stimulated polarized B cells. We then targeted 233 candidates in a siRNA screen and identified hits regulating the polarization of the centrosome and/or lysosomes in B cells upon stimulation. Our dataset of proteomics, images, and polarity indexes provides a valuable source of information for a broad community of scientists interested in the molecular mechanisms regulating cell polarity.

## Background & Summary

Cell polarization is instrumental in a broad range of biological processes, from the cellular scale, regulating cell migration, cell division and cell communication^1,2^, to the tissue and organism levels, controlling tissue organization and functions^3–5^. One central event in cell polarization is the relocation of the centrosome at one pole of the cell, establishing the cell polarity axis^6^. This notably applies to B lymphocytes^7^, which therefore constitute a model of choice for the study of cell polarity. To fulfill their immune function, B lymphocytes need to recognize their specific antigen to become activated and proceed further with antibody production. In brief, engagement of the B cell receptor (BCR) with antigens immobilized at the surface of an antigen-carrying cell induces the formation of an immune synapse at the contact site. This first event of symmetry breaking triggers the repositioning of the centrosome and lysosomes toward the immune synapse^8^. Lysosomes are then secreted within the synaptic space, allowing antigen extraction and internalization for further presentation to specific CD4^+^ T cells, which provide the secondary signals required for antigen-specific antibody production^9^.

Interestingly, we recently found that the centrosome proteome changes upon B-cell stimulation with surface-tethered antigens^10^. We therefore asked whether the proteins differentially associated with the centrosome between resting and antigen-stimulated polarized B-lymphocytes are required or not for B cell polarization. To this end, we developed a medium-throughput screening strategy based on siRNA-mediated silencing of individual proteins, automated cell imaging, and analysis of centrosome and lysosome polarity. Since polarity pathways are highly conserved between various cell types and across species^7^, we believe that the proteins identified through this screen performed on B cells might reveal common players involved in cell polarization processes, which will be of interest for a broad community of scientists.

**Fig. 1:**
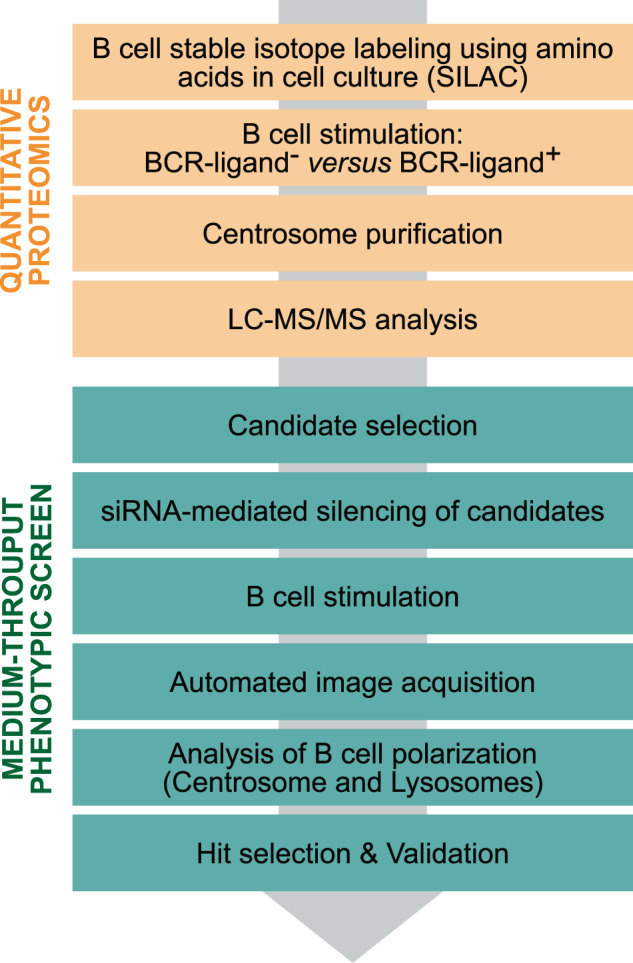
Overview of the screening strategy. A SILAC-based quantitative proteomics was first performed on purified centrosomes from resting non-polarized and stimulated polarized B cells to identify candidates potentially involved in B cell polarization. The role of these candidates in the polarization of centrosome and/or lysosomes was then tested in a medium-throughput image-based phenotypic siRNA screen. Automated image analysis revealed hits, some of which were further validated.

In this study, we first established a list of proteins differentially associated with the centrosome between resting and polarized states in B cells. We wished to start with a large list of candidates. To this end, we built up on our previous study by performing two additional replicates of stable isotope labelling by amino acids in cell culture (SILAC)-based quantitative proteomics on purified centrosomes and completed this list with interesting candidates from the literature. We then selected 233 proteins and tested their implication in centrosome and/or lysosome polarization. To do so, we individually silenced these proteins with SmartPool siRNA, stimulated B cells with antigen-coated beads for 90 min, fixed and stained the cells for automated fluorescence imaging, and finally assessed centrosome and lysosome polarity using an in-house developed Fiji macro. Some of the selected hits that affected centrosome and/or lysosome polarization were already known regulators of cell polarity from the literature, confirming the robustness of our approach and dataset (Fig. 1).

## Methods

### B lymphoma cell line

For this study, we used the mouse IgG^+^ B-lymphoma cell line IIA1.6, a commonly used model of B cells. This cell line is derived from the A20 cell line (ATCC #TIB-208) and present the advantage of being easily electroporated for siRNA delivery. IIA1.6 cells were cultured in CLICK medium (RMPI 1640- Glutamax-I, 10% Fetal Calf Serum, 1% Penicillin/Streptomycin, 0.1% β-mercaptoethanol, 2% sodium pyruvate) and maintained at 37 °C in a humidified atmosphere with 5% CO_2_. Cells were centrifuged at 1200 rpm (340 g) for 5 min at room temperature and sub-cultured every 2-3 days at 1:4. Cells were routinely tested for the absence of mycoplasma contamination.

All experiments were performed using 50% CLICK/50% RPMI 1640-Glutamax-I. All cell culture products were purchased from Gibco LifeTechnologies.

### Primary B cells

Primary B cells were purified from spleens of Chmp2b^+/+^ and Chmp2b^−/−^ mice using mouse negative B cell isolation kit (Miltenyi) according to manufacturer’s instructions. These mice were previously generated by genetrap insertion in the intron between exons 2 and 3 of the Chmp2b gene (chromosome 16; 16 C1.3)^11^. Founders were kindly provided by A. Isaacs and bred in our SPF animal facility as Chmp2b^+/−^ male crossed with Chmp2b^+/−^ females. Littermate controls were used in each experiment. Experiments and sacrifices were performed in accordance with the guidelines and regulation of the French veterinary department and the ethical committee of the Institut Curie and strictly conformed to the European and French National Regulation for the Protection of Vertebrate Animals used for Experimental and other Scientific Purposes (Directive 2010/63; French Decree 2013-118). All experiments were then performed using 50% CLICK/50% RPMI 1640-Glutamax-I. All cell culture products were purchased from Gibco LifeTechnologies.

### Identification of centrosome-associated proteome by SILAC analysis

In order to extend the initial list of proteins differentially associated with the centrosome of resting non-polarized and antigen-stimulated polarized B cells, we repeated the SILAC-based quantitative proteomics (reverse-phase liquid chromatography and high-resolution mass spectrometry) analysis on centrosomes purified from both conditions, as previously described^10,12^. Three technical replicates, including two forward- and one reverse-cell labelling, were thus performed and analyzed together. For replicates #1 and #3 (“forward”) we used a light isotope (^12^C_6_) of lysine to label non-polarized B cells (incubated with non-stimulating beads) and a heavy isotope (^13^C_6_) to label polarized B cells (incubated with stimulating beads). In the “reverse” experiment (replicate #2), and to rule out any effect of isotope incorporation, we used “light” ^12^C_6_-labelled B cells for quantifying centrosome-associated proteins in polarized B cells and “heavy” ^13^C_6_-labelled B cells for quantifying centrosome-associated proteins in non-polarized B cells. For each replicate, purified centrosomes from polarized and non-polarized B cells were mixed to a 1:1 ratio, analyzed by LC-MS/MS and the relative quantification of the centrosome-associated proteins was performed by computing the “Heavy/Light” ratio (replicates #1 and #3) or “Light/Heavy” (replicate #2) for each of the identified proteins in each replicate.

For LC-MS/MS analysis, proteins were separated on 10% SDS-PAGE gels, reduced (10 mM DTT), alkylated (55 mM iodoacetamide) and then digested overnight with endoproteinase rLys-C (Promega). Peptides were then extracted and analyzed by nano-LC-MS/MS using an Ultimate 3,000 system (Dionex S.A.) coupled to an LTQ-Orbitrap XL mass spectrometer (Thermo Fisher Scientific). SILAC-based protein quantification used peptide XICs (extracted ion chromatograms) retrieved from Proteome Discoverer. Scale normalization computed using the ‘package limma’ from R was applied to compensate for mixing errors of the different SILAC cultures.

**Fig. 2:**
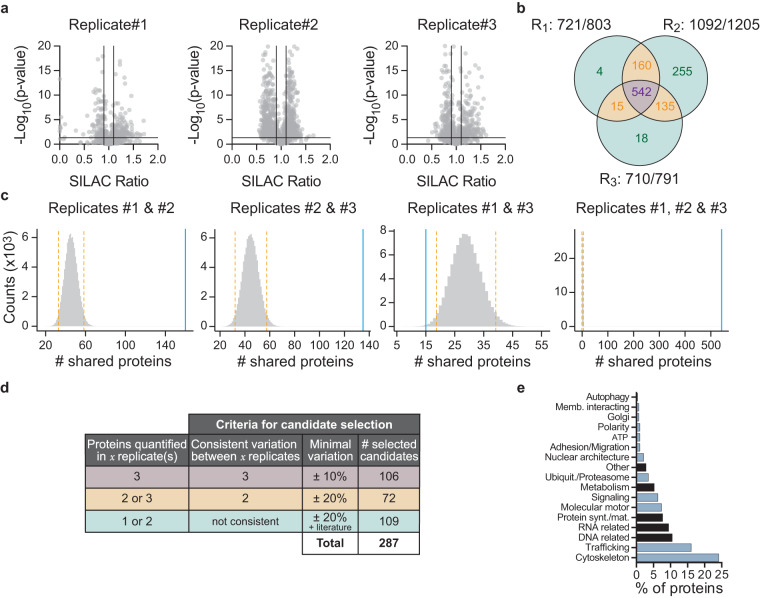
Candidate selection from the SILAC-based quantitative proteomic analysis. (**a**) Volcano-plots of -log_10_ (p-value) as a function of the SILAC ratio per replicate. The vertical lines represent the first threshold used for biological variation (± 10%). The horizontal lines represent the threshold used for statistical significance (p-value ≤ 0.05). (**b**) Venn diagram of the number of proteins identified in each replicate as varying (absolute variation ≥10%, and p-value ≤ 0.05). (**c**) Histograms of the simulated distribution of shared proteins between replicates as estimated by the *in-silico* permutation test. Orange dashed vertical lines show ± 2 standard deviations from the mean. The blue vertical line in each graph shows the observed number of shared proteins in our experimental dataset for the corresponding comparison. (**d**) Criteria for candidate selection. (**e**) Functional classification of candidates using UniProtKB. Selected candidates are highlighted in blue.

The relative quantification of proteins between the resting non-polarized and antigen-stimulated polarized conditions was then performed. Protein ratios were calculated as the geometrical mean of related peptides. To estimate ratio significance, a t-test was performed with a Benjamini–Hochberg FDR control threshold. Based on three criteria (number of peptides used ≥ 3, absolute fold change ≥ 1, and adjusted p-value ≤ 0.05), we formally identified 1,253 proteins out of a total of 2,915 quantified proteins (Fig. 2a), among which 1,129 proteins showed an absolute variation ≥10%. 852 of them were found in at least two replicates (Fig. 2b). We next used an *in-silico* permutation test to estimate whether this overlap was one could anticipate by chance. We simulated 3 replicates by uniformly sampling 721; 1,092; and 710 proteins (corresponding to the numbers of selected proteins in replicate #1, #2, and #3, respectively, Fig. 2b) among the 16,547 proteins found in the SwissProt *mus musculus* (version 20120905) database used to identified proteins following the mass spectrometry analysis. Such a simulation was repeated 100,000 times and the distribution of the numbers of shared proteins between replicates (one-by-one, and between the three) was then computed. Strikingly, this procedure showed that the numbers of shared proteins observed experimentally (Fig. 2b) fall into low probability areas (Fig. 2c), strongly arguing for a non-random overlap between replicates in our dataset. Similar conclusions were obtained using the SuperExactTest function of the R package^13^. The final selection of proteins differentially associated with the centrosome for the phenotypic screen was based on the number of replicates in which they were quantified and their extent of variation (See below).

### Selection of candidates for the phenotypic screen

In order to maximize the potential discovery of unexpected regulators of centrosome repositioning, differential criteria with relatively low stringency were applied to select candidate proteins. Therefore, we first selected 106 proteins showing consistent variation in the three replicates and a variation of at least ± 10% in one of them. We completed with 72 proteins harboring consistent variation of at least ± 20% in two replicates. To these 178 proteins, 109 proteins that displayed non-consistent variation between replicates or identified only in one replicate were added according to their described function in the literature and a minimal variation of ± 20%, bringing the number of selected proteins to 287 (Fig. 2d). The functional classification of these 287 proteins was then performed using the UniProtKB database (Fig. 2e) and we focused our study on the 187 proteins known to regulate organelle positioning, intracellular trafficking and signaling as well as cell adhesion and migration (Fig. 2e, blue bars). This primary list was then complemented by 46 proteins described in the literature to regulate cell polarity and/or cytoskeleton dynamics. Overall, this selection strategy led to the inclusion of 233 candidate proteins (See Data Records).

### siRNA-mediated silencing of candidates

We next individually silenced these 233 proteins. To do so, we decided to use the ON-TARGETplus SmartPool siRNA technology from Dharmacon. One advantage of this strategy (over classical single oligo siRNA) is to lower the off-target rate, which is key to obtain reliable results in a screen. The off-target effects of siRNA are mainly due to the high concentration needed to achieve efficient silencing as well as the intrinsic chemical properties of the oligos^14,15^. In the ON-TARGETplus SmartPool strategy, the specificity of silencing is ensured by decreasing by 4-fold the relative concentration of each oligo sequence and modifying their chemistry^16^, while a high silencing efficiency is maintained by pooling 4 different oligos against the gene of interest. Therefore, 1.10^6^ IIA1.6 cells were electroporated with 200 nM ON-TARGETplus SmartPool siRNA (Dharmacon custom library) against individual candidates, using the Neon electroporation system (10 µL tips, 1300 V, 2 pulses, 20 ms.pulse^−1^, Invitrogen, Life Technologies). Non-targeting SmartPool siRNA were used as control (pool of 4 siRNAs designed and validated by the manufacturer for minimal off-target of mouse genes^17^). Electroporated cells were seeded in cell-culture treated 24-well plates (1.10^6^ cells/1.5 mL of CLICK-medium) and incubated for 72 h at 37 °C, 5% CO_2_. Efficiency of silencing was assessed by Western blot on a specific target present on all plates (Myh9, see *Technical Validation*).

### Preparation of stimulating beads

To mimic surface-tethered antigen recognition, we used beads coated with anti-BCR as a surrogate for antigen-carrying cell, as previously described^9,10^. Stimulating beads were prepared 24 h prior to use. 84.10^6^ 3 µm-polystyrene NH_2_-beads (50 µL of stock, Polyscience, #17145-5) were activated with 500 µL 8% Glutaraldehyde for 3 h at room temperature on a rotating wheel. Beads were then centrifuged at 13 000 rpm for 10 min on a tabletop centrifuge, washed twice with 1 mL of PBS, resuspended in 400 µL of anti-BCR containing solution (Goat F(ab’)_2_ anti-mouse IgG, MP Biomedicals, final concentration 0.1 µg.µL^−1^, referred to as BCR-ligand^+^) or a control solution (Goat F(ab’)_2_ anti-mouse IgM, MP Biomedicals, same concentration, referred to as BCR-ligand^−^) and incubated overnight on a rotating wheel at 4 °C. Beads were washed 3 times with 1 mL of PBS just before use, and resuspended at 1.6.10^6^ beads.mL^−1^ in 50% CLICK/50% RPMI 1640-Glutamax-I. This bead suspension was sufficient to test simultaneously the 233 candidates and include control wells in 96-well plates, at a 1:2 cell:bead ratio, with 1.10^5^ cells per well.

### Cell stimulation and immunofluorescence

96-well plates were coated with 50 µL of Poly-L-lysine (Sigma Merck, # P8920) solution per well for 3 h at room temperature, washed 3 times with 200 µL of distilled water, and dried for 2 h in a 37 °C oven.

Cells were harvested, counted, and assessed for viability using trypan Blue, then resuspended in 50% CLICK/50% RPMI 1640-Glutamax-I at 2.10^6^ cells.mL^−1^ and seeded at 1.10^5^ cells per well (50 µL). Cells were let to sediment for 15 min, before adding 2.10^5^ anti-BCR coated beads (125 µL) and incubated 90 min at 37 °C, 5% CO_2_. Supernatant was then removed gently, cells were gently washed once with 50 µL of PBS and fixed for 2 min on ice with 50 µL of cold methanol (conserved at −20 °C until use). Cells were gently washed again with 200 µL of PBS and permeabilized/saturated in 50 µL of PBS-BSA 0.2%-Saponine 0.05% for 10 min at room temperature. Liquid was gently removed, and cells were incubated with primary antibodies diluted in PBS-BSA-Saponin for 45 min at room temperature. Centrosomes were stained using Rabbit anti-γ-tubulin (Abcam, #Ab11317, 1:2000) and lysosomes with Rat anti-Lamp-1 (CD107a, BD Pharmingen, #553792, 1:400). Cells were washed twice with 200 µL of PBS and incubated for 30 min at room temperature in the dark with the following secondary antibodies (all from Jackson ImmunoResearch, 1:200) diluted in PBS-BSA-Saponin: AffiniPure F(ab’)_2_ AlexaFluor488-conjugated Donkey Anti-Rabbit IgG (#711-546-152), Cyanine3-conjugated AffiniPure F(ab’)_2_ Donkey Anti-Rat IgG (#712-166-150), and AlexaFluo647-conjugated AffiniPure F(ab’)_2_ Donkey Anti-Goat IgG (#705-606-147) to stain the beads. Nuclei were counterstained using DAPI, and cells were kept at 4 °C in 1% Paraformaldehyde until imaging.

### Image acquisition and processing

**Fig. 3:**
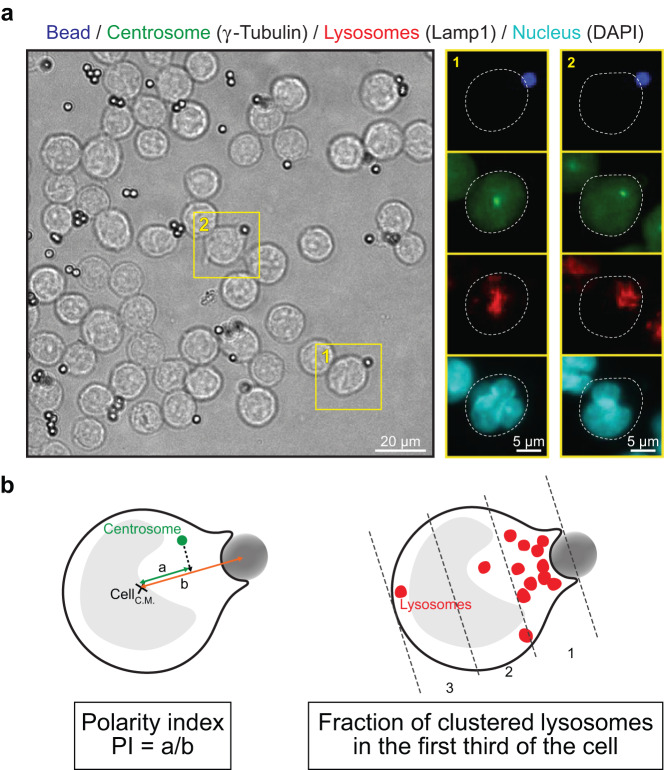
Image analysis pipeline and definition of polarity indexes. (**a**) Representative wide-field image of B cells stimulated with antigens immobilized at the surface of beads. Inserts show zooms on B cell-bead pairs stained for IgG (bead), γ-Tubulin (centrosome), and Lamp1 (lysosomes). Nuclei were counterstained with DAPI. (**b**) Schematics depicting the calculation of centrosome and lysosome polarity indexes.

Images were acquired on a high content imaging system (ImageXpress Micro, Molecular Devices, Sunnyvale, USA) with a Nikon (Tokyo, Japan) S-Fluor 20x, 0.75 NA objective. 11 positions were acquired in each well to maximize the area covered to account for intra-well heterogeneity (Fig. 3a). Focus was automatically detected based on DAPI fluorescence and one focal plane was acquired per position.

Images were then analyzed automatically using in-house Fiji macros to determine centrosome and lysosome polarity indexes. For each well, cells and beads were segmented, and bead−cell pairs were selected and cropped (Macro 1). Big clusters of cells or beads were removed by size-thresholding, and a watershed was applied to individualize touching cells. Bead−cell pairs were identified as overlapping segmentation masks from the two corresponding fluorescence channels. Finally, only bead−cell pairs with a polarity axis in the focal plane were kept by thresholding on the distance between bead center of mass and cell center of mass (>0.5 radius of the cell). Polarity indexes were then measured for each bead−cell pair (Macro 2). For centrosome polarity index, centrosome position was detected and projected on the axis determined by the center of mass of the cell mask and the center of mass of the bead mask obtained from Macro 1. The centrosome polarity index was then defined as the distance (a) between the projected centrosome and the center of mass of the cell divided by the distance (b) between the center of mass of the cell and the center of mass of the bead (Fig. 3b, left). A polarity index equal to 1 reflects a full polarization toward the immune synapse, 0 indicates non-polarized state, −1 anti-polarized. The lysosome polarity index was calculated as the fraction of total lysosome-associated fluorescence intensity located in the first third of the cell in contact with the bead (Fig. 3b, right). To do so, images were rotated to be aligned on the bead-cell axis, background was subtracted, and fluorescence intensity was quantified in the whole cell (F_T_) or in the first third of the cell in contact with the bead (F_B_). Lysosome polarity index was computed as the ratio F_B_/F_T_. A polarity index of 1 reflects a full polarization toward the immune synapse, while 0.3 corresponds to a non-polarized dispersed state. Finally, results were compiled and normalized to the median polarity value of the reference well (B01, siCtrl) for each plate (Macro 3).

### Hit selection

**Fig. 4:**
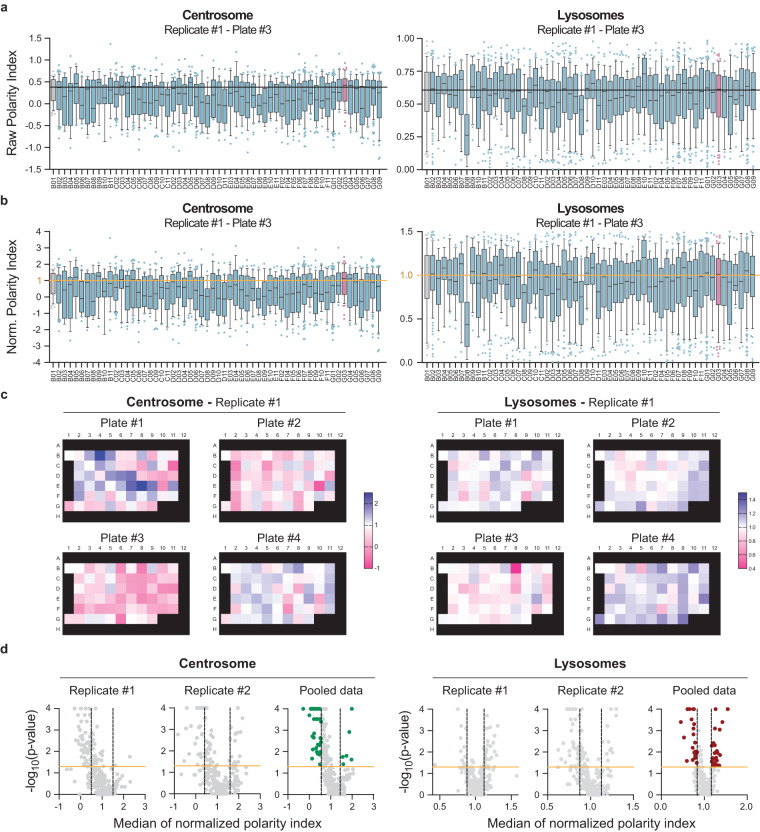
Identification of hits from the siRNA screen. (**a,b**) Representative raw and normalized polarity indexes from individual cells of one plate (Plate #3, Replicate #1). Left: Centrosome. Right: Lysosomes. Boxes in box plots extend from the 25^th^ to the 75^th^ percentiles, with a line at the median, whiskers extend from the 10^th^ to the 90^th^ percentiles, and individual dots denote outlier values. (**c**) Median values of normalized polarity indexes per condition per plate, presented as the 96-well plate layout of the screen. Data presented are from the 4 plates of replicate #1. Left: Centrosome. Right: Lysosomes. (**d**) Volcano-plots of -log_10_ (p-value) as a function of the median values of normalized polarity indexes per condition per plate and per replicate (#1 and #2), as well as pooled data. Vertical lines represent the threshold used for biological variation (± 1 SD). Horizontal lines represent the threshold used for statistical significance (p-value ≤ 0.05, Wilcoxon Signed-Rank test). Colored symbols correspond to selected hits.

After the calculation of raw polarity indexes for individual cells in each well (as illustrated for plate #3 from replicate #1 in Fig. 4a), the corresponding normalized polarity indexes were calculated by dividing them by the median value of the control well (B01) per plate and replicate (Norm. PI_B01_ = 1, Fig. 4b). Figure 4c provides an overview of the median of the normalized polarity indexes of all wells in the 4 plates of replicate #1. Normalization per plate and per replicate allowed us to pool, for each candidate, the individual normalized polarity indexes of all cell-bead couples from replicates #1 and #2 before performing hit selection. The median was then recalculated for each candidate on the pooled dataset (Fig. 4d). Proteins were considered as hits when their silencing significantly decreased or increased centrosome or lysosome repositioning more than one standard deviation (1 SD) from the control condition in the pooled data (Fig. 4d). SD was calculated as the standard deviation of the distribution of the medians of the normalized pooled polarity indexes. This strategy led to a hit discovery rate of about 20%, with some hits exclusively altering the polarization of the centrosome or lysosomes, while some affected both. Of note, since the silencing efficiency has not been assessed for each of the individual candidate genes following siRNA treatment, we cannot exclude a potential role of proteins showing no effect on the polarization of the centrosome and/or the lysosome. All statistical analyses were performed using GraphPad Prism v9.1.0 (216).

## Data Records

The mass spectrometry proteomics raw data files (.raw), the Mascot merged result files (.msf) used for data analysis in myProMS^18^ and a table summarizing the full list of proteins differentially associated with centrosome between resting non-polarized and stimulated polarized B cells identified by SILAC-based quantitative proteomics (absolute fold change ≥ 1 AND p-value ≤ 0,05 AND peptide used ≥ 3) have been deposited to the ProteomeXchange Consortium^19^ via the PRIDE partner repository^20^ with the dataset identifier PXD038725^21^. The dataset is organized as such:

- 3 excel files (Design PRIDE 20 fractions R1; Design PRIDE 17 fractions R2; and Design PRIDE 30 fractions R3) summarizing the correspondence between the gel slices and the associated raw files per replicate. Briefly, F6804FD.raw to F6806FD.raw and F6843FD.raw to F6859FD.raw correspond to the 20 raw files of replicate #1; F7195FD.raw to F7211FD.raw to the 17 raw files of replicate #2; and F6002FD.raw to F6031FD.raw to the 30 raw files of replicate #3.
- The 67 raw files (20 for replicate #1, 17 for replicate #2, and 30 for replicate #3) that contain the spectra from each run. The correspondence of each raw file is specified in the 3 excel files named “Design PRIDE…”, as described above.
- 3 Mascot merged result files (F6859FD.msf; F7211FD.msf; and F6031FD.msf) used to process the shotgun MS/MS data through the sequence search engine SEQUEST to identify MS/MS spectra based on a list of protein sequences for replicate 1#; #2; and #3, respectively.
- An excel file (Proteomics_data_and_selected_candidates) summarizing all identified and quantified proteins and the one selected as candidates for the siRNA-based screen.

Images of the siRNA screen are publicly available in the BioStudies database under the accession number S-BSST897^22^ (https://identifiers.org/biostudies:S-BSST897).

The data is organized as such:

- An excel file (Submission-File_list) providing all the information on each image: Localization in the zip file, Name of the image, Replicate, Plate, Well, Field (position of imaging), Target gene name, NCBI gene ID, Description (control or target), QC info, Channels identity.
- A zip file containing all the images, named as listed in the excel file Submission-File_list. Images are organized in folders per replicate and per plate. The identity of each image file is specified in its name, according to the Submission_File-list. For example, Rep3-Plate4-Well_B01-Pos8 corresponds to the Replicate #3, Plate 4, Well B01, position 8, i.e. silencing of ACTR3 (line 7,291 of Submission-File_list).
- An excel file (cells_beads_pairs_count) of the numbers of cells, beads, and cell-bead pairs in each well of each plate and per replicate.
- An excel file (single_pairs_Raw_and_Normalized_polarity_indexes) of the raw and normalized polarity indexes of individual cell-bead pairs for all silencing conditions tested per plate and replicate. One tab corresponds to one plate (P) of one replicate (R) for centrosome (Cent) or lysosomes (Lyso).
- An excel file (Median_Normalized_Polarity_Indexes) summarizing the median values of normalized polarity indexes and associated statistics per silencing condition and replicate for centrosome and lysosomes.
- An excel file (Screen_Hits) summarizing the proteins selected as hits after the screen.

## Technical Validation

### Efficiency of silencing

**Fig. 5:**
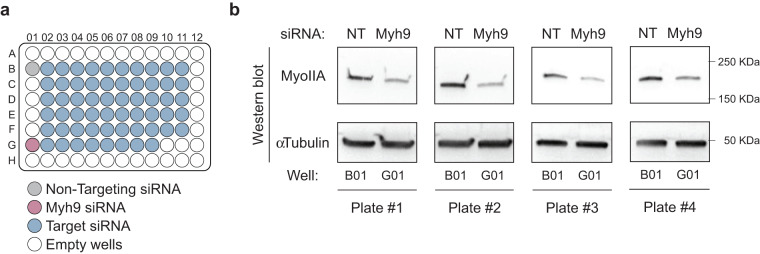
Validation of silencing efficiency. (**a**) siRNA screen plate layout (identical for the 4 plates of each replicate). (**b**) Representative Western blots against Myosin IIA and α-Tubulin (loading control) on control cells (non-targeting (NT) siRNA) and Myosin IIA-silenced cells (Myh9 siRNA) from replicate #1.

Given the number of conditions and replicates, validating the efficiency of silencing for each well was not manageable. To validate the electroporation and the efficiency of silencing for each plate and each replicate, we therefore performed a Western blot on control cells (non-targeting siRNA, B01 well) and one silenced condition (siMyh9, G01 well) per plate and per replicate (Fig. 5a). αTubulin was used as a loading control. In brief, B cells were lysed at 4 °C in RIPA buffer (Thermo Scientific) supplemented with 1x protease inhibitor cocktail (Roche) and 1x Halt phosphatase inhibitor cocktail (Thermo Scientific). Supernatants were collected and loaded onto mini-PROTEAN TGX SDS–PAGE gels and transferred onto polyvinylidene fluoride membrane (Trans-Blot Turbo Transfer). Membranes were blocked in 5% non-fat dry milk resuspended in 1x TBS–0.05% Tween-20 and incubated overnight at 4 °C, with primary antibodies (anti-myosin IIA Heavy chain, Covance, 1:1000 and anti-αTubulin, Serotec, 1:1000) followed by 60 min incubation with HRP-conjugated secondary antibodies. Western blots were developed with Clarity Western ECL substrate, and chemiluminescence was detected using the ChemiDoc imager (all from BioRad). Western blot showed efficient silencing of Myh9 in all plates of all replicates (Fig. 5b). We assumed silencing was achieved in every other condition as well.

### Validation of automated analysis of centrosome and lysosome polarity

**Fig. 6:**
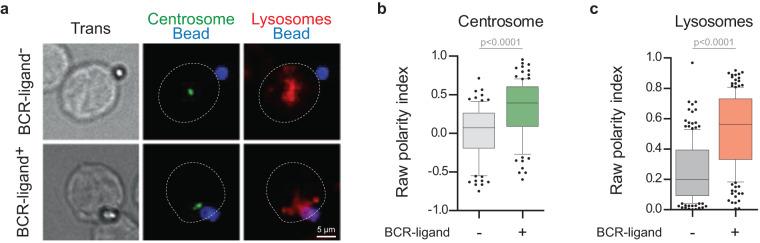
Validation of the automated image analysis. (**a**) Representative images of control non-electroporated cells in contact with non-stimulating beads (BCR-ligand^−^) or stimulating beads (BCR-ligand^+^) in the same imaging conditions as the screen. B cell-bead pairs were stained for IgG (bead), γ-Tubulin (centrosome), and Lamp1 (lysosomes). (**b,c**) Centrosome and Lysosome raw polarity indexes from individual cells. Boxes in box plots extend from the 25^th^ to the 75^th^ percentiles, with a line at the median, whiskers extend from the 10^th^ to the 90^th^ percentiles, and individual dots denote outlier values. Kolmogorov-Smirnov test was used to assess the normal distribution of the dataset. Mann-Whitney test was used to assess statistical significance.

To validate the in-house Fiji macro that calculates polarity indexes, we acquired images on a “Control plate” that included 10 wells of non-electroporated cells in contact with non-stimulating beads (BCR-ligand^−^) and 10 wells of non-electroporated cells in contact with stimulating beads (BCR-ligand^+^) in the same imaging conditions as the screen (Fig. 6a). Images were then analyzed with our in-house macros, statistical analyses were performed using GraphPad Prism v9.1.0 (216). For centrosome, resting B cells (BCR-ligand^−^ condition) displayed polarity indexes close to 0, while activated cells (BCR-ligand^+^ condition) showed a median polarity index of 0.4 (Fig. 6b), reflecting the centrosome polarization induced by BCR stimulation. For lysosomes, resting B cells (BCR-ligand^−^ condition) displayed polarity indexes close to 0.3, corresponding to a third of lysosomes in the first third of the cell in contact with the bead, *i.e*. non-polarized state. Activated cells (BCR-ligand^+^ condition) showed a median lysosome polarity index of 0.6 (Fig. 6c), i.*e*. 2/3 of their lysosomes were clustered in the first third of the cell in contact with the bead, confirming their polarization toward the stimulating bead. This analysis on control non-electroporated cells confirmed that our method is suitable to analyze our screen and efficiently discriminate polarizing conditions from non-polarizing conditions.

### Screening quality check

**Fig. 7:**
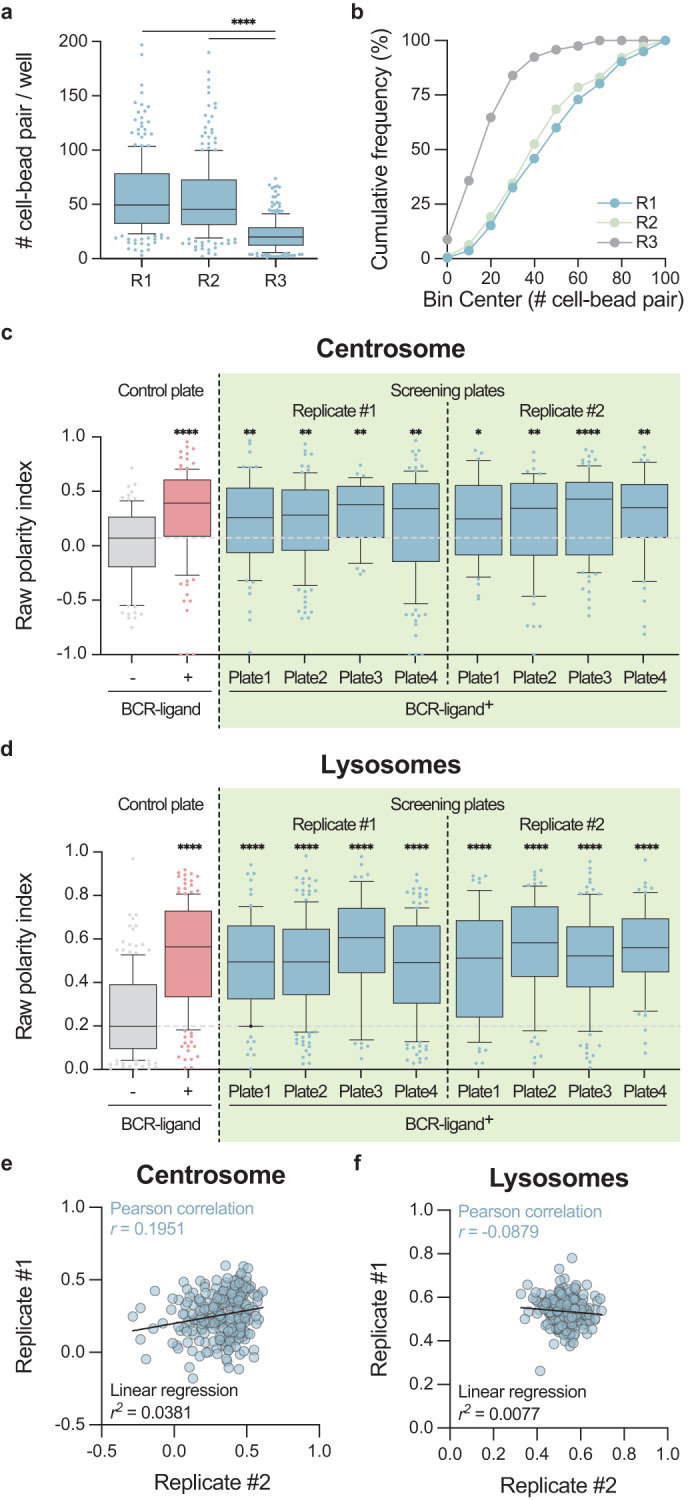
Screen quality check. (**a**) Numbers of cell-bead pairs per well and replicate. Boxes in box plots extend from the 25^th^ to the 75^th^ percentiles, with a line at the median, whiskers extend from the 10^th^ to the 90^th^ percentiles, and individual dots denote outlier values. Kolmogorov-Smirnov test was used to assess the normal distribution of the dataset. Kruskal-Wallis with Dunn’s correction for multiple comparisons was used to assess statistical significance. (**b**) Cumulative frequency in percentage of the numbers of cell-bead pairs per well in each replicate. (**c,d**) Centrosome and lysosome raw polarity indexes of non-targeting siRNA-treated B cells (well B01, light blue) incubated with stimulating beads per plate and replicate and compared to the extent of polarization observed in non-electroporated control B cells incubated with either stimulating (light red) or non-stimulating (grey) beads. Boxes in box plots extend from the 25^th^ to the 75^th^ percentiles, with a line at the median, whiskers extend from the 10^th^ to the 90^th^ percentiles, and individual dots denote outlier values. Kolmogorov-Smirnov test was used to assess the normal distribution of the dataset. Kruskal-Wallis with Dunn’s correction for multiple comparisons was used to assess statistical significance. (**e,f**) Correlation analysis of centrosome and lysosome median polarity indexes in replicate #1 *versus* replicate #2.

To validate the plate design strategy and the overall quality of the screen, we first checked the number of cell-bead pairs per well identified automatically by Macro 1. While the number of pairs were similar in replicates #1 and #2, it was significantly lower in replicate #3 (Fig. 7a,b). We thus decided to exclude replicate #3 for the hit selection.

To assess the robustness and the per plate reproducibility of our assay, we included in each plate of each replicate a negative siRNA control (non-targeting siRNA, well B01) for which cells were incubated with stimulating beads. This well provided a positive control of both centrosome and lysosome polarization for each plate, which could be then compared to the extent of polarization observed in the “Control plate” (of non-electroporated cells, Fig. 6). As shown in Fig. 7c,d, the polarization of both the centrosome and the lysosomes was not affected by the non-targeting siRNA. Indeed, their polarity indexes were similar to the ones of non-electroporated cells in contact with stimulating beads (BCR-ligand^+^), but significantly higher than the polarity indexes of non-electroporated cells in contact with non-stimulating beads (BCR-ligand^−^). This strongly suggests that our experimental approach is sensitive and robust enough to discriminate potential hits affecting centrosome and/or lysosome polarization upon their silencing using our SmartPool siRNA strategy.

Finally, we evaluated the reproducibility of the two selected replicates, by testing the correlation of the median value of raw polarity indexes between the two replicates for each siRNA tested. Because the variability between the two replicates was high (Fig. 7e,f), and because the aim of our screen was to identify potential regulators of cell polarity, we decided to pool the normalized polarity indexes of the two replicates to take into account this variability in our subsequent selection. Yet, all the polarity indexes are available per replicate^22^, should a future study wish to use a different selection strategy.

All statistical analyses needed for the quality check were performed using GraphPad Prism v9.1.0 (216).

### Validation of hits

**Fig. 8:**
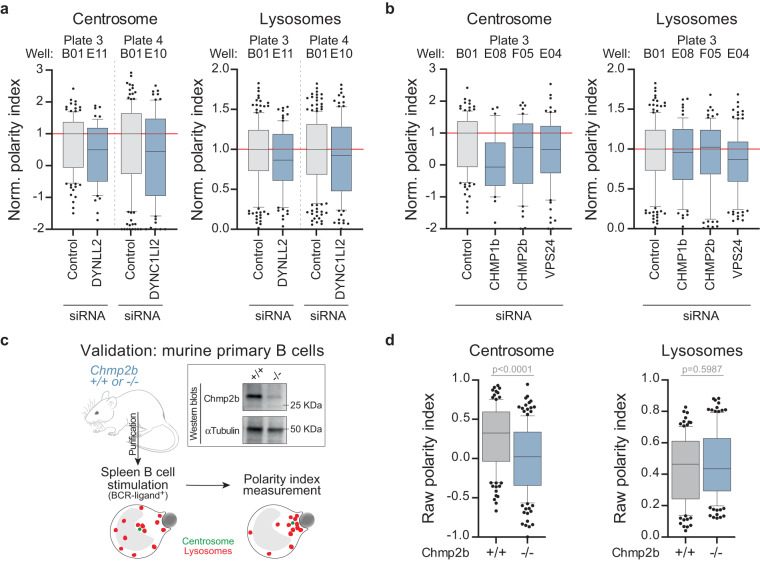
Validation of the screening strategy. (**a,b**) Centrosome and lysosome normalized polarity indexes extracted from the screening dataset for the selected hits previously described in the literature as regulators of cell polarity (Dynein and ESCRT-III subunits, respectively). (**c**) Primary B cells were purified from the spleens of Chmp2b^+/+^ and Chmp2b^−/−^ mice and stimulated with BCR-ligand^+^ beads. Expression of Chmp2b was verified by Western blot. (**d**) Centrosome and lysosome polarity indexes from Chmp2b^+/+^ and Chmp2b^−/−^ primary B cells stimulated with BCR-ligand^+^ beads. Boxes in box plots extend from the 25^th^ to the 75^th^ percentiles, with a line at the median, whiskers extend from the 10^th^ to the 90^th^ percentiles, and individual dots denote outlier values. Kolmogorov-Smirnov test was used to assess the normal distribution of the dataset. Mann-Whitney test was used to assess statistical significance.

Our screen identified essential regulators of centrosome and/or lysosome polarization upon B cell stimulation with surface-tethered antigens. Among these hits, some were already known regulators from the literature, such as Dynein subunits^8^ (Fig. 8a) and multiple subunits of the endosomal sorting complex required for transport (ESCRT)-III complex (Chmp1b, Chmp2b and Vps24)^23,24^ (Fig. 8b). Proteins from the ESCRT machinery are well known to regulate endo-lysosome biogenesis, membrane repair, as well as centrosome maintenance^25–27^. Since our siRNA-mediated screen was performed on IIA1.6 B lymphoma cell line, our strategy may present two caveats: the use of immortalized cell lines, and the efficiency/specificity of the siRNA silencing. To rule out these possibilities, we compared the polarity observed after silencing of Chmp2b in the screen to the polarity induced by stimulating-beads in primary B cells purified from the spleens of Chmp2b knock-out (Chmp2b^−/−^) mice^11^, hence lacking completely Chmp2b expression. These were compared to their wild-type (Chmp2b^+/+^) counterparts. Experiments on primary cells were consistent with the observations made in the screen: Chmp2b is necessary for the proper polarization of centrosome but dispensable for lysosome polarization (Fig. 8c,d). Taken together, these observations validate our primary screening strategy of using siRNA-silenced B cell lymphoma cell line to identify polarity regulators. A secondary screen would be required for the definitive validation of the selected hits.

## Data Availability

Annotated macros used to analyze the screen are available on GitHub: https://github.com/Mathieu-Maurin/screening_B_cell_polarization. The files are organized as such: - README.md: This file contains brief instructions on how to use the macros. - Macro_1_cell_bead_couple_extraction: The purpose of this macro is to extract bead/cell couples for analysis. It works on composite images reconstructed with the following channels: Channel 1: gamma-Tubulin; Channel 2: Dapi; Channel 3: Lamp1; Channel 4: Fluorescent beads. - Macro_2_polarity_analysis: The purpose of this macro is to determine centrosome and lysosome raw polarity indexes for each bead/cell couple extracted by Macro_1. - Macro_3_normalise_and_concatenate_tables. The purpose of this macro is to normalize data and concatenate tables. It works after Macro 2. Output tables are in the format PlateName-Well_A01_Results. A01 indicates the well coordinate in the plate. It contains all the polarity results from all positions of a given well. The macro concatenates and normalizes data to get a single table for centrosome-polarity or lysosomes-polarity. It computes also median values for each well. Normalization is based on the median value of well B01 of each plate (non-targeting siRNA), which is normalized to 1. - Macro_4_count_cell_beads_and_couple. The purpose of this macro is to control bead, cell, and couple counts. It works after Macro 3 and adds a result table in the final table folder.
